# Comparison of Transcriptional Changes to Chloroplast and Mitochondrial Perturbations Reveals Common and Specific Responses in Arabidopsis

**DOI:** 10.3389/fpls.2012.00281

**Published:** 2012-12-24

**Authors:** Olivier Van Aken, James Whelan

**Affiliations:** ^1^ARC Centre of Excellence in Plant Energy Biology, University of Western AustraliaCrawley, WA, Australia

**Keywords:** *Arabidopsis*, mitochondria, chloroplasts, retrograde, stress, microarray

## Abstract

Throughout the life of a plant, the biogenesis and fine-tuning of energy organelles is essential both under normal growth and stress conditions. Communication from organelle to nucleus is essential to adapt gene regulation and protein synthesis specifically to the current needs of the plant. This organelle-to-nuclear communication is termed retrograde signaling and has been studied extensively over the last decades. In this study we have used large-scale gene expression data sets relating to perturbations of chloroplast and mitochondrial function to gain further insights into plant retrograde signaling and how mitochondrial and chloroplast retrograde pathways interact and differ. Twenty seven studies were included that assess transcript profiles in response to chemical inhibition as well as genetic mutations of organellar proteins. The results show a highly significant overlap between gene expression changes triggered by chloroplast and mitochondrial perturbations. These overlapping gene expression changes appear to be common with general abiotic, biotic, and nutrient stresses. However, retrograde signaling pathways are capable of distinguishing the source of the perturbation as indicated by a statistical overrepresentation of changes in genes encoding proteins of the affected organelle. Organelle-specific overrepresented functional categories among others relate to energy metabolism and protein synthesis. Our analysis also suggests that WRKY transcription factors play a coordinating role on the interface of both organellar signaling pathways. Global comparison of the expression profiles for each experiment revealed that the recently identified chloroplast retrograde pathway using phospho-adenosine phosphate is possibly more related to mitochondrial than chloroplast perturbations. Furthermore, new marker genes have been identified that respond specifically to mitochondrial and/or chloroplast dysfunction.

## Introduction

It is well established that the energy organelles chloroplasts and mitochondria communicate with the cellular nucleus to allow for optimal functioning through signaling processes generally termed retrograde signaling. One reason for this retrograde regulation is easily envisaged, as during evolution of eukaryotes the majority of genes encoding plastid or mitochondrial proteins have relocated to the nucleus (Bock and Timmis, 2008). Therefore, the organelles must feed back to the nucleus to induce or reduce transcription of organellar proteins depending on developmental cues and environmental conditions. Accordingly, several nuclear marker genes encoding organellar proteins have been used in the past to study retrograde signaling, including *LHCB2.4* for plastid retrograde (Koussevitzky et al., 2007) and *AOX1a* for mitochondrial retrograde signaling (Dojcinovic et al., 2005; Giraud et al., 2009). When looking at the co-expression of genes encoded in the nucleus and genes still encoded in the organellar genome, it was found that co-expression could be observed for nuclear and organellar genes encoding plastid proteins, but not mitochondrial proteins (Leister et al., 2011).

However, it is apparent from a number of studies that these retrograde signals do not just affect the expression of genes encoding proteins targeted to the specific organelle, but also other cellular compartments and functional groups (Van Aken et al., 2007; Estavillo et al., 2011; Giraud et al., 2012). Recent studies have indicated for instance that mitochondrial perturbations can significantly affect functional categories such as plant-pathogen interactions, protein synthesis, and photosynthetic light reactions (Schwarzlander et al., 2012). In the case of mitochondrial retrograde signaling, links, and overlaps have also been shown with stress-responses and resistance (Rhoads and Subbaiah, 2007; Van Aken et al., 2007). Other studies have suggested that many signaling pathways are overlapping and very hard to separate, resulting in a lack of marker genes encoding chloroplast or mitochondrial proteins that respond only to specific conditions (Leister et al., 2011).

Although some insight has been developed regarding the transcriptional responses ensuing retrograde signaling, very few transcriptional regulators have been identified. For mitochondrial retrograde signaling, so far only Abscisic acid Insensitive 4 has been identified as a regulator, and interestingly ABI4 had also been identified as an important regulator of chloroplast retrograde signaling (Koussevitzky et al., 2007; Giraud et al., 2009). Other proteins involved in chloroplast retrograde signaling contain the Genomes Uncoupled (GUN) proteins that act during early development of chloroplast function termed biogenic control (Susek et al., 1993; Koussevitzky et al., 2007) but are very divergent types of proteins ranging from pentatricopeptide repeat (PPR) protein GUN1 to Mg-chelatase H (Mochizuki et al., 2001; Koussevitzky et al., 2007). The actual molecules that may be messenger of GUN signals are controversial, with some groups suggesting Mg-protoporphyrin IX and other groups contesting this (Mochizuki et al., 2008; Moulin et al., 2008).

Once functional chloroplasts have been established, other retrograde pathways have been identified that regulate chloroplast function termed operational control. For example, during drought and high-light conditions a signaling pathway is active via SAL1, involved in dephosphorylation of phospho-adenosine phosphate (PAP), and XRN-type exoribonucleases that are inhibited by PAP concentrations (Chen et al., 2011; Estavillo et al., 2011). Another class of likely signals for chloroplast and probably mitochondrial retrograde signaling are reactive oxygen species (ROS). ROS are readily produced in metabolically active cells both in chloroplasts and mitochondria (among other subcellular sites), with the four common forms of ROS generated being singlet oxygen (^1^O_2_), the superoxide anion $\left({\text{O}}_{\text{2}}^{-}\right)$, hydrogen peroxide (H_2_O_2_), and the hydroxyl radical (HO•). ROS formation has also been long recognized as a common signal in all kinds of biotic and abiotic stresses, malnutrition, and hormone signaling. The most broadly studied type of ROS is probably H_2_O_2_, and it is a good candidate for involvement in retrograde signaling as it is a relatively stable molecule and could potentially travel from the chloroplasts or mitochondria to the cytosol or nucleus (Chan et al., 2010). Also singlet oxygen is thought to play a role in chloroplast retrograde signaling, as evidenced in the *fluorescent* (*flu*) mutant that accumulates protochlorophyllide in the chloroplasts during dark periods, and subsequently generates singlet oxygen once illuminated resulting in genome-wide expression changes and eventually cell death (Meskauskiene et al., 2001; Lee et al., 2007). Elimination of the Executer 1 protein is able to suppress these cell death signals in the *flu* mutant. Nevertheless, given the short life-span of ROS and the myriad of processes that can increase their presence, they are difficult to study specifically in the context of retrograde signaling.

To gain more detailed insight into the transcriptional effects that are common and different between chloroplast and mitochondrial retrograde signaling pathways we collected a series of microarray experiments that examined perturbations in either system. Most studies have employed chemical inhibitions using inhibitors such as norflurazon, lincomycin for chloroplasts and rotenone, oligomycin, and antimycin A for mitochondria. These compounds are known to have indirect side-effects that make interpretation more complicated. Therefore, several mutants in specifically chloroplast of mitochondrial proteins were examined. Furthermore, the expression of genes that responded significantly to these organellar perturbations was examined across a wide variety of developmental and stress-related microarray experiments to allow a better picture of how retrograde responses are situated within the lifecycle of a plant.

## Materials and Methods

### Generation of organelle-specific gene lists

Genes encoding chloroplast, mitochondrial or peroxisome proteins were selected based on publicly available information based on mass-spectrometry, Green Fluorescent Protein-targeting assays, and subcellular localization prediction algorithms. The list for mitochondria was taken from a previous study (Law et al., 2012). For chloroplasts, the *Arabidopsis* subcellular database SUBA (Heazlewood et al., 2007) was used as basis and genes for which either experimental (GFP targeting or more than half of mass-spectrometry identifications) or predicted (five or more of the 10 prediction algorithms) localization in chloroplasts was evidenced were selected. Furthermore, this list was expanded with the plant proteomics database PPDB (Sun et al., 2009) and was further expanded with chloroplast protein identifications published by (Yu et al., 2008). In this way 2384 genes were selected and in total 2183 probe sets represented genes in this list on the Affymetrix ATH1 GeneChip microarrays. Similarly, SUBA was used as a starting point for peroxisomal proteins and genes for which predicted (two or more of the 10 prediction algorithms) localization in peroxisomes was evidenced were selected. Proteins reported by several proteomic studies were also included (Reumann et al., 2007, 2009; Eubel et al., 2008; Lingner et al., 2011), as well as the Peroxisomes 2010^1^ list of confirmed peroxisomal proteins. In total 306 genes encoding peroxisomal proteins were selected and in total 287 probe sets represented genes in this list on the Affymetrix ATH1 GeneChip. The list of transcription factors was based on their presence in at least two of the four *Arabidopsis* transcription factor databases RARTF, AGRIS, PlnTFDB, and DATF (Mitsuda and Ohme-Takagi, 2009), and as such 1983 genes encoding putative transcription factors. In total 1661 probe sets represented genes in this list on the Affymetrix ATH1 GeneChip. All gene lists are shown in Table S1 in Supplementary Material.

### Microarray data analysis

For the datasets related to chloroplast and mitochondrial perturbations CEL files were normalized using the MAS5.0 algorithm within the Avadis microarray analysis software package. Probe sets that were not called absent in at least half of the chips for one genotype or treatment were kept for further analysis. Statistical analysis was then performed using the CyberT Bayesian framework (Baldi and Long, 2001) and genes with average foldchange of more than twofold and PPDE (<*p*) > 0.95 were retained. If probe sets were called absent an expression value of fold change 1 was assigned for further analysis. Complete expression and statistical analysis results are available in Table S2 in Supplementary Material. CEL files for the additional microarray experiments related to stress, development, anatomy, nutrient availability etc. were also normalized using the MAS5.0 algorithm and probe sets that were not called absent in at least half of the chips for one timepoint, treatment or tissue type were kept for further analysis. If probe sets were called absent an expression value of fold change 1 was assigned for further analysis. Normalized expression data for all probes in all selected experiments are available in Table S3 in Supplementary Material. Visualization and hierarchical clustering of the microarray data were performed in the Multiexperiment Viewer MeV 4.8^2^ using the Pearson correlation coefficient. Overrepresentation of functional categories was performed using the PageMan tool (Usadel et al., 2006). Functional categories with significant over- or underrepresentation (*p* < 0.05) and a ratio foldchange of twofold or more were retained. Complete PageMan outputs are shown in Table S4 in Supplementary Material.

### Promoter motif analysis

The 1 kb promoter regions of the selected marker genes were downloaded from TAIR^3^. The MEME suite was then used to discover overrepresented motifs in an unbiased search (Bailey et al., 2009). MEME parameters used were any number of motif repetitions per sequence, minimum motif width of five bases and maximum motif width of 50 bases. For analysis of known binding sites the Athena promoter analysis tool was used (O’Connor et al., 2005).

## Results

### Selection of microarray datasets

First, public repositories were searched for available microarray experiments that directly affect mitochondrial function in *Arabidopsis thaliana* (Table 1). For normalization purposes and comparability only datasets based on the Affymetrix ATH1 platform were selected, as this is by far the most widely used system for analysis of *A. thaliana* gene expression. Studies using mitochondrial inhibitor compounds included treatment with rotenone (Complex I inhibitor), oligomycin (ATP synthase inhibitor; Clifton et al., 2005), and antimycin A (Complex III inhibitor; Ng et al., 2012, GEO accession GSE36011). Additionally, several mutants in genes encoding mitochondrial proteins have been studied using microarrays. These include prohibitin *atphb3* (Van Aken et al., 2007), Complex I subunits *ndufs4*, and *ndufa1* (Meyer et al., 2009), alternative oxidase *aox1a* (Giraud et al., 2008), organellar RNA polymerase *rpotmp* (Kuhn et al., 2009), succinate dehydrogenase subunit *dsr1* (Gleason et al., 2011), regulators of mitochondrial recombination *msh1 recA3* double mutant (Shedge et al., 2010), mitochondrial splicing factor *rug3* (Kuhn et al., 2011), mitochondrial inner membrane translocase subunit *Tim23-2* knockout and overexpressor (Wang et al., 2012) and mitochondrial translation factor double mutant *letm1(*−/−*) LETM2(*+/−; Zhang et al., 2012). In total these comprise 14 different perturbations of mitochondrial function.

**Table 1:** **List of microarray experiments related to mitochondrial and chloroplast perturbations used in this study**.

	Experimental conditions	Source
**MITOCHONDRIAL PERTURBATIONS**		
Rotenone	40 μM, cell culture, 3 h	Clifton et al. (2005)
Oligomycin	12.5 μM, cell culture, 3 h	Clifton et al. (2005)
Antimycin A	50 μM, 3-week-old leaves in light, long day, 3 h	Ng et al. (2012) GEO GSE36011
*atphb3*	*In vitro* seedlings growth stage 1.04	Van Aken et al. (2007)
*ndufs4*	Leaves harvested in middle of photoperiod	Meyer et al. (2009)
*ndufa1*	Leaves harvested in middle of photoperiod	Meyer et al. (2009)
*aox1a*	4-week-old soil-grown plants	Giraud et al. (2008)
*rpotmp*	4-week-old soil-grown plants	Kuhn et al. (2009)
*dsr1*	10 h after treatment with water (mock)	Gleason et al. (2011)
*msh1 recA3*	Above-ground parts of 8 week old plants	Shedge et al. (2010)
*rug3*	*In vitro* seedlings 7 days old	Kuhn et al. (2011)
*tim23-2*	16-day-old leaf tissue	Wang et al. (2012)
*TIM23-2 OX*	16-day-old leaf tissue	Wang et al. (2012)
*letm1*(−/−) *LETM2*(+/−)	10-day-old leaf tissue	Zhang et al. (2012)
**CHLOROPLAST PERTURBATIONS**		
Norflurazon (Col-0, *gun1 gun5*)	3-day-old *in vitro* seedlings 5 μm	E-GEOD-5726
Lincomycin (Col-0, *abi4-102*, *gun1 gun5*)	5-day-old *in vitro* seedlings	Koussevitzky et al. (2007)
PNO8	1 μM 7-day-old *in vitro* seedlings	Goda et al. (2008)
*fnr1*	31-day-old plants middle of photoperiod	Lintala et al. (2009)
*cs26*	3-week-old leaves, long day	Bermudez et al. (2010)
*tpt-1*	Leaves growth stage 3.7; 2 h into photoperiod, 400 μE	E-GEOD-5737
*flu*	3-week-old rosette leaves 2 h reillumination	Laloi et al. (2007)
*35S:tAPX*	3-week-old rosette leaves 2 h reillumination	Laloi et al. (2007)
*sal1*	Whole rosette from seedlings stage 1.10	Estavillo et al. (2011)
*xrn2 xrn3*	Whole rosette from seedlings stage 1.10	Estavillo et al. (2011)

Next a similar selection of microarray experiments directly affecting chloroplast function and chloroplast retrograde regulation was made (Table 1). These include inhibitor studies using chlorophyll synthesis inhibitor norflurazon (McCormac and Terry, 2004), photosystem II protein synthesis inhibitor lincomycin (Koussevitzky et al., 2007), photosystem II inhibitor PNO8 (Goda et al., 2008). In addition the *gun1*, *gun5*, and *abi4* mutants in chloroplast retrograde signaling were compared against Col-0 wild type plants after treatment with lincomycin (Koussevitzky et al., 2007). Finally a number of mutants in genes encoding chloroplast proteins were selected, including leaf-type ferredoxin-NADP+ oxidoreductase *fnr1* (Lintala et al., 2009), S-sulfocysteine synthase *cs26* (Bermudez et al., 2010), as well as three mutants in chloroplast proteins that display distinct responses under high-light conditions including triose-phosphate translocator *tpt-1* (Walters et al., 2004), the *flu* mutant that accumulates the photosensitizer protochlorophyllide in the dark, and the thylakoid ascorbate overexpressor *35S:tAPX* (Laloi et al., 2007). Lastly, the *sal1* mutant in a protein dual-targeted to plastids and mitochondria that is involved in dephosphorylation of PAP was included. Accumulated PAP in *sal1* can relocate from plastids to cytosol and nucleus and triggers a retrograde signaling pathway from chloroplast to nucleus, possibly through PAP-induced inactivation of XRN-type exoribonucleases. Both *sal1* and *xrn2 xrn3* mutants were included (Estavillo et al., 2011).

Thirdly, a collection of publicly available microarray datasets encompassing various aspects of *Arabidopsis* development and environmental responsiveness were put together. For anatomy and development these included the AtGenExpress development dataset with over 60 tissue-types and developmental stages from embryogenesis to senescence (Schmid et al., 2005), as well as the radial root cell-type specific expression dataset (Brady et al., 2007), germination timecourse (Narsai et al., 2011), 24 h diurnal expression (Arrayexpress experiment E-GEOD-6174) and cell cycle synchronization with aphidicolin, sucrose deprivation and subculturing (Menges et al., 2003). For hormone response the AtGenExpress hormone datasets for auxin, cytokinin, gibberellin, brassinosteroid, abscisic acid, jasmonate, and ethylene (Goda et al., 2008) was used in addition to salicylic acid treatment (Clifton et al., 2005). For abiotic stress the AtGenExpress datasets for heat, salt, UV, genotoxic stress, oxidative stress, and osmotic stress (Kilian et al., 2007) were collected in addition to hydrogen peroxide (Clifton et al., 2005), high-light (Kleine et al., 2007), and ozone (Short et al., 2012). For biotic stresses the AtGenExpress datasets for infection with *Botrytis cinerea*, *Phytophtora infestans*, *Erysiphe orontii*, and elicitor Flg22 were selected (Goda et al., 2008), as well as infection with *E. cichoracearum* (Nishimura et al., 2003), *Blumeria graminis* (Jensen et al., 2008), and elicitor EF-Tu (Zipfel et al., 2006). For nutrient deprivation the following experiments were selected: iron deficiency (Schuler et al., 2011), potassium deprivation (E-GEOD-6825), nitrogen deprivation (Rubin et al., 2009), phosphate starvation (Li et al., 2010), sulfur deprivation (Iyer-Pascuzzi et al., 2011), exogenous sucrose addition (E-NASC-29), and high CO_2_ (E-GEOD-5637).

### Perturbation of mitochondrial and chloroplast function triggers overlapping but targeted expression changes

**Figure 1:**
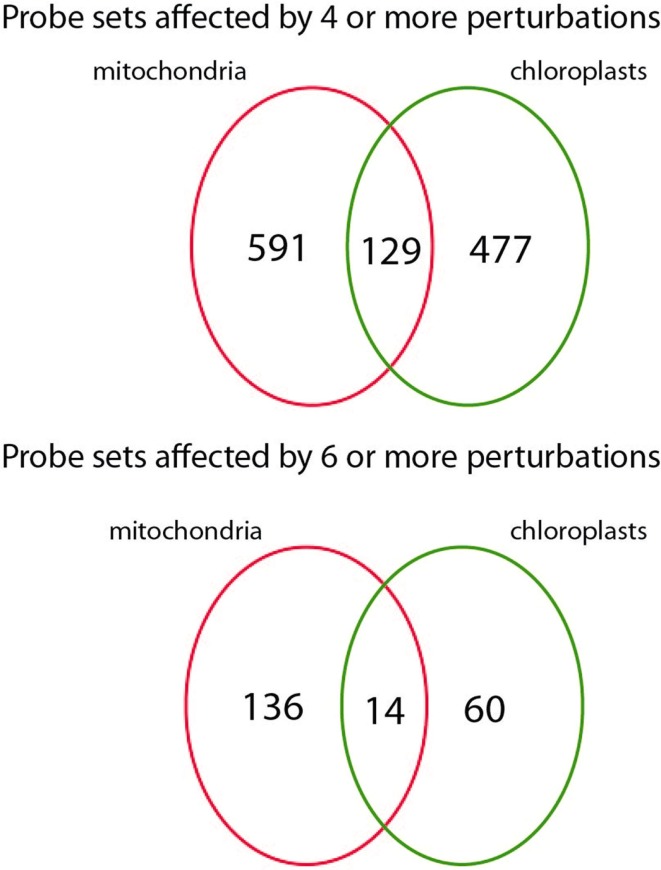
**Number ATH1 microarray probe sets responding to mitochondrial and chloroplast perturbations**. Number of probe sets that were significantly altered in at least four (top) or six (bottom) microarray experiments relating to mitochondrial or chloroplast perturbations and their overlap.

To assess the transcriptional changes that are caused by directly perturbing mitochondrial or chloroplast function, the selected microarray datasets were normalized and statistically analyzed. Genes that were significantly changed [PPDE (<*p*) > 0.95] and had a fold change of more than two up or down were selected for each of the microarray experiments described above (Table 2). For the mitochondrial perturbations 720 probe sets were significantly changed in four or more of the 14 mitochondrial perturbation conditions. For the treatments or mutations that affected chloroplast function or retrograde signaling 606 probe sets were altered significantly in four or more of 13 chloroplast perturbation conditions. When comparing the two datasets it was found that 129 probe sets were in common between mitochondrial and chloroplast perturbations. This indicates an early sevenfold overabundance of common genes compared to the expected 19 in a random distribution (*p* < 0.001) and shows that perturbation of chloroplast or mitochondrial function results in surprisingly similar transcriptional responses (Figure 1).

**Table 2:** **Perturbation of mitochondrial and chloroplast function triggers overlapping but targeted expression changes**.

**(A)**						
	Mitoch. perturb.	Chloroplast perturb.	Observed overlap	Expected	*p*-Value	Obs/exp
Chloroplast and mitochondrial overlap	720	606	129	19	<0.001	6.74
**(B)**						
	Compartment	Genome-wide	Observed	Expected	*p*-Value	Obs/exp
Chloroplast perturbations (606)	Chloroplasts	2198	177	58	<0.001	3.03
	Mitochondria	1178	31	31	>0.05	0.99
	Peroxisome	287	7	8	>0.05	0.92
	Transcription factors	1661	41	44	>0.05	0.93
Mitochondrial perturbations (720)	Chloroplasts	2198	81	70	>0.05	1.16
	Mitochondria	1178	88	38	<0.001	2.34
	Peroxisome	287	4	9	>0.05	0.44
	Transcription factors	1661	71	53	<0.01	1.34

*(A) Chi-Squared statistical analysis of the number of overlapping probe sets changing in at least four experiments perturbing mitochondrial or chloroplast function. Observed and expected overlaps, Chi-squared *p*-value and ratio of observed overlap versus expected overlap (obs/exp) are shown. Abbreviations: mitoch, mitochondrial; perturb, perturbation. (B) Chi-squared statistical analysis of the number of probe sets per subcellular compartment and transcription factors responding to chloroplast and mitochondrial perturbations. Shown are the number of probes in the entire genome (genome-wide) per compartment, total number of probes responding to at least four chloroplast or mitochondrial perturbations (606 and 720 probe sets, respectively), observed and expected number of responsive probes per compartment, Chi-squared *p*-value and ratio of observed overlap versus expected overlap (obs/exp)*.

The next step was to assess whether a bias could be observed with respect to which energy organelle was being target by the expression changes. Therefore, comprehensive lists were made of genes encoding mitochondrial (1178), chloroplast (2198), or peroxisomal proteins (287) based on experimental and prediction information (see Materials and Methods). Also a list of 1661 *Arabidopsis* transcription factors was prepared. Table 2 shows the observed and expected number of probe sets representing energy organelle proteins that were present in the mitochondrial (720 probe sets) and chloroplast (606) perturbation data sets. Interestingly, a statistically significant overrepresentation of probe sets was shown that specifically represent proteins of the organelle where the initial perturbation originated, but not the other energy organelles. In other words, mitochondrial perturbation triggered significantly more expression changes in genes encoding mitochondrial proteins than randomly expected (*p* < 0.001), but not in genes encoding chloroplast or peroxisome proteins (*p* > 0.05). Conversely, chloroplast perturbation triggered significantly more expression changes in genes encoding chloroplast proteins than randomly expected (*p* < 0.001), but not in genes encoding mitochondrial or peroxisome proteins (*p* > 0.05). Furthermore, mitochondrial perturbations triggered statistically more changes in expression of transcription factors than randomly expected (*p* < 0.01), indicating an extensive network of transcriptional regulation underlies these expression changes. In conclusion, this analysis revealed that despite the large overlap between expression changes triggered by mitochondrial and chloroplast dysfunction, retrograde signals coming from chloroplasts or mitochondria are able to at least partially distinguish the signal source and can effectuate a targeted response.

Subsequently, the responses to chloroplast or mitochondrial perturbations were analyzed in more detail with regards to overrepresentation of functional classes. Therefore, the PageMan analysis tool was used to analyze the genes that were changed in expression due to chloroplast or mitochondrial perturbations. The expression values of all probe sets that were significantly changed in at least four of the mitochondrial or chloroplast perturbations were selected, resulting in 720 and 606 probe sets, respectively. Functional categories that were significantly overrepresented or underrepresented with *p*-value below 0.05 and greater than twofold change compared to the control group are shown in Table 3. For mitochondrial perturbations, several overrepresented categories are mitochondria and carbohydrate metabolism-related, such as alternative oxidases and NADH dehydrogenases, ATP synthase, cytochrome c oxidase, as well as fermentation and prokaryotic-like (mitochondrial) ribosomal proteins. Interestingly, in our study the photosynthesis functional category was underrepresented during mitochondrial perturbations, whereas another study found photosynthesis as being overrepresented (Schwarzlander et al., 2012). This difference may be caused by the use of different sets of microarray studies and a different analysis algorithm (Pageman versus Mapman). Abiotic and oxidative stress categories were also overrepresented, including heat stress, glutaredoxins, and glutathione *S*-transferases. Protein turnover was also present with AAA-type proteases, protein folding, and prokaryotic ribosomal proteins overrepresented, while ubiquitin-type degradation was underrepresented. Strikingly, auxin- and ethylene metabolism were statistically overrepresented, as well as several transcription factor classes including WRKY, pseudoresponse ARR, MYB, Constans, and pbf2.

**Table 3:** **Significantly over- or underrepresented functional categories among probe sets commonly responding to mitochondrial (top) or chloroplast (bottom) dysfunction as determined by PageMan analysis**.

BIN	Bin name	Counted	Genome	*p-*Value	Ratio
**MITOCHONDRIAL PERTURBATIONS**					
5.3	Fermentation.ADH	1	1	0.0320	31.25
25.1	C1-metabolism.formate dehydrogenase	1	1	0.0320	31.25
27.3.82	RNA.regulation of transcription.plant TF (pbf2)	1	1	0.0320	31.25
9.2.2	Mitochondrial electron transport/ATP synthesis.NADH-DH external	2	4	0.0059	15.62
9.4	Mitochondrial electron transport/ATP synthesis.alternative oxidase	2	5	0.0096	12.50
11.9.4.13	Lipidmetabolism.lipid degradation.beta-oxidation.acyl CoA reductase	2	6	0.0141	10.42
27.3.66	RNA.regulation of transcription.Pseudo ARR transcription factor family	2	8	0.0252	7.81
24.2	Biodegradation of Xenobiotics	2	10	0.0388	6.25
23.1.2	Nucleotide metabolism.synthesis.purine	3	15	0.0111	6.25
12	N-metabolism	4	24	0.0066	5.21
27.3.7	RNA.regulation of transcription, Constans-like zinc finger family	4	25	0.0077	5.00
21.4	Redox.glutaredoxins	5	33	0.0037	4.73
29.6	Protein.folding	9	62	0.0001	4.54
15.2	Metal handling.binding, chelation, and storage	6	44	0.0026	4.26
3.2	Minor CHO metabolism.trehalose	3	22	0.0320	4.26
9.9	Mitochondrial electron transport/ATP synthesis.F1-ATPase	3	22	0.0320	4.26
30.2.17	Signaling.receptor kinases.DUF 26	6	44	0.0026	4.26
26.9	Glutathione *S*-transferases	7	53	0.0014	4.13
28.1.3	DNA.synthesis/chromatin structure.histone	6	46	0.0033	4.08
9.7	Mitochondrial electron transport/ATP synthesis.cytochrome c oxidase	3	23	0.0359	4.08
23.1	Nucleotide metabolism.synthesis	4	32	0.0184	3.91
17.5.2	Hormone metabolism.ethylene	4	35	0.0248	3.57
9	Mitochondrial electron transport/ATP synthesis	14	124	0.0000	3.53
27.3.26	RNA.regulation of transcription.MYB-related transcription factor family	4	40	0.0383	3.12
29.5.9	Protein.degradation.AAA-type	4	41	0.0414	3.05
27.3.32	RNA.regulation of transcription.WRKY domain transcription factor family	6	62	0.0142	3.02
17.2	Hormone metabolism.auxin	14	152	0.0004	2.88
13.2	Amino acid metabolism.degradation (glutamate, GABA)	6	67	0.0202	2.80
2	Major CHO metabolism	8	99	0.0141	2.52
20.2.1	Stress.abiotic.heat	12	151	0.0034	2.48
29.2.1.1	Protein.synthesis.ribosomal protein.prokaryotic	10	127	0.0077	2.46
16.8	Secondary metabolism.flavonoids	6	82	0.0476	2.29
10	Cell wall	33	471	0.0000	2.19
34.16	Transport.ABC transporters and multidrug resistance systems	8	115	0.0315	2.17
29.5.11	Protein.degradation.ubiquitin	12	988	0.0001	0.38
1	PS photosystems	1	187	0.0336	0.17
**CHLOROPLAST PERTUBATIONS**					
19.16	Tetrapyrrole synthesis.chlorophyll b synthase	1	1	0.0266	37.54
16.8.2.1	Secondary metabolism.flavonoids.chalcones.naringenin-chalcone synthase	1	1	0.0266	37.54
13.2.2.2	Amino acid metabolism.degradation.glutamatefamily.proline	2	3	0.0021	25.02
19.14	Tetrapyrrole synthesis.protochlorophyllide reductase	2	3	0.0021	25.02
11.1.11	Lipid metabolism, FA synthesis, and FA elongation.fatty acid elongase	2	5	0.0067	15.01
1.1	PS.light reaction	49	136	0.0000	13.52
1	PS	59	187	0.0000	11.84
13.1.5.1	Amino acid metabolism.synthesis.serine-glycine-cysteine group.serine	2	7	0.0136	10.72
1.3	PS.calvincyle	7	31	0.0000	8.48
20.2.2	Stress.abiotic.cold	4	20	0.0017	7.51
21.4	Redox.glutaredoxins	6	33	0.0002	6.82
17.8	Hormone metabolism.salicylic acid	3	19	0.0133	5.93
33.1	Development.storage proteins	4	26	0.0047	5.77
2.1.2	Major CHO metabolism.synthesis.starch	4	26	0.0047	5.77
14	S-assimilation	2	13	0.0455	5.77
1.2	PS.photo-respiration	3	20	0.0153	5.63
19	Tetrapyrrole synthesis	6	45	0.0012	5.00
21.2.1	Redox.ascorbate and glutathione.ascorbate	3	24	0.0251	4.69
33.2	Development.late embryogenesis abundant	3	26	0.0310	4.33
2.2.2	Major CHO metabolism.degradation.starch	3	26	0.0310	4.33
26.9	Misc.glutathione S-transferases	6	53	0.0028	4.25
29.2.1.1.1	Protein.synthesis.ribosomalprotein.prokaryotic.chloroplast	7	68	0.0022	3.86
17.5	Hormone metabolism.ethylene	9	93	0.0008	3.63
27.3.32	RNA.regulation of transcription.WRKY domain transcription factor family	6	62	0.0061	3.63
16.5.1	Secondary metabolism.sulfur-containing.glucosinolates	5	54	0.0143	3.48
17.3	Hormone metabolism.brassinosteroid	4	49	0.0412	3.06
29.6	Protein.folding	5	62	0.0246	3.03
17.1	Hormone metabolism.abscisic acid	4	50	0.0438	3.00
20.2.1	Stress.abiotic.heat	12	151	0.0007	2.98
15	Metal handling	5	64	0.0278	2.93
10.7	Cellwall.modification	5	66	0.0312	2.84
17.2	Hormone metabolism.auxin	11	152	0.0026	2.72
21	Redox	13	190	0.0018	2.57
26.1	Misc.cytochrome P450	13	199	0.0027	2.45
29.5.11	Protein.degradation.ubiquitin	13	988	0.0045	0.49
29.4	Protein.postranslational modification	6	668	0.0020	0.34
28.1	DNA.synthesis/chromatin structure	1	753	0.0000	0.05

*Counted: number of members per functional category in specific probe list; Genome: number of members per functional category genome-wide; *p*-value: statistical significance; ratio: foldchange of number of members per functional category compared to the expected random distribution*.

For chloroplast perturbations, as expected photosynthesis-related categories were overrepresented including photosystems, Calvin cycle, photo-respiration, and tetrapyrrole (chlorophyll B and protochlorophyllide) synthesis. DNA synthesis category was downregulated, where it was upregulated in mitochondrial perturbations. Several similarities could also be observed between chloroplast and mitochondrial perturbations, mainly including redox (glutaredoxins, glutathione *S*-transferases), abiotic stress and protein turnover/degradation, but also metal handling, starch metabolism and cell wall structuring. The only overrepresented transcription factor family was the WRKY family, which interestingly was also overrepresented during mitochondrial perturbation. Several hormone related categories were overrepresented including salicylic acid, auxins, ethylene, brassinosteroids, and abscisic acid.

### Comparison of mitochondrial and chloroplast perturbations provides evidence for *sal*1 and *xrn*2 *xrn*3-mediated PAP pathway in mitochondrial retrograde regulation

**Figure 2:**
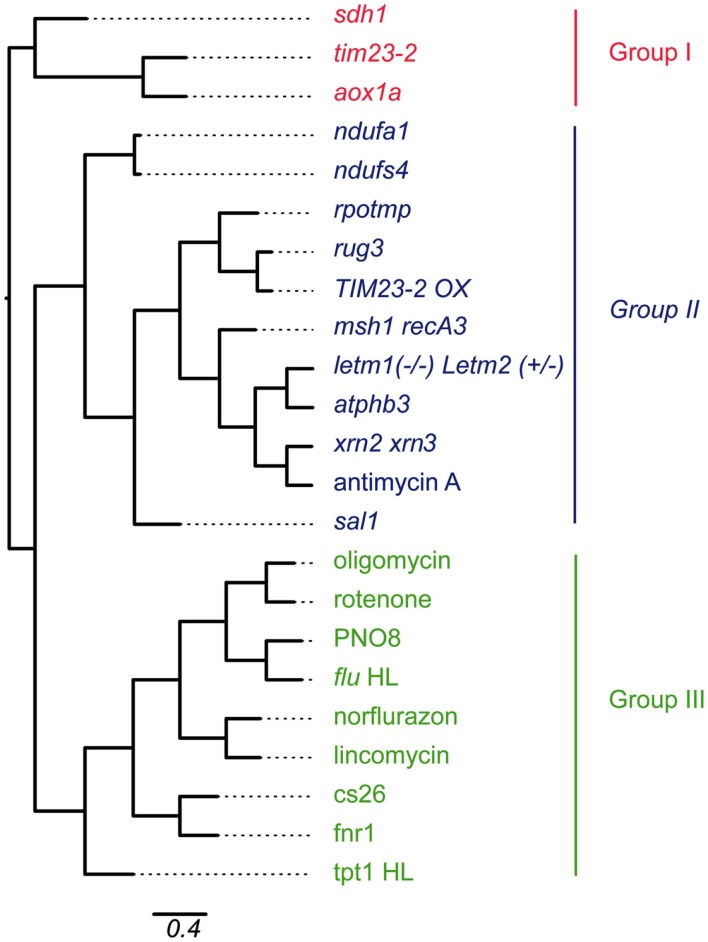
**Hierarchical clustering of microarray experiments relating to mitochondrial and chloroplast perturbations**. Gene expression values of probe sets that were significantly altered in at least four of 13 experiments relating to mitochondrial or chloroplast perturbations were hierarchically clustered using Pearson correlation coefficient. Branch lengths indicate relative distance in expression patterns as indicated by the scale bar (percentage identity). Abbreviations: Cell cul, cell culture; Chloro Pert, chloroplast perturbation; Mito pert, mitochondrial perturbation.

Given the wide range of perturbations of mitochondrial and chloroplast function through mutation or chemical inhibition, it is of interest to examine which of these conditions show similarity with each other. Therefore, a hierarchical clustering method was applied on the expression values of a selection of the mitochondrial and chloroplast perturbation microarray experiments (Figure 2).

The expression values of all probe sets that were significantly changed in at least four of the mitochondrial or chloroplast perturbations were pooled, resulting in 1197 probe sets. The sample sets were hierarchically clustered using Pearson correlation with average linkage and visually represented as a phylogenetic tree (Figure 2). The clustering approach demonstrated the presence of three main groups of expression patterns. The co-clustering of specific perturbations appears to be mostly independent of tissue-types and ages used for the microarray experiments (Table 1). Looking at the samples present in each group revealed a number of expected patterns, but also some unexpected patterns were observed. Group I contains a number of mitochondrial mutants including *aox1a*, *tim23-2*, and Complex II subunit *sdh1*. These mitochondrial mutants clustered separately from the other mitochondrial mutants in Group II. Group II contained the remaining mutants in mitochondrial proteins with a variety of functions. Two subgroups were evident, one containing complex I subunit mutants *ndufa2* and *ndufs4*. The second subgroup contained splicing factor mutant *rug3*, *TIM23-2* overexpressor, and dual-targeted *RNA polymerase rpotmp*, all of which are known to be indirect Complex I mutants. The second subgroup also contained prohibitin mutant *atphb3*, mitochondrial genome recombination mutant *msh1 recA3*, mitochondrial mutant *letm1(*−/−*)* *Letm2(*+/−*)*, and chemical inhibitor of Complex III antimycin A. Surprisingly, this subgroup also contained the mutant in a recently described chloroplast retrograde pathway *sal1* revolving around PAP and the nuclear exoribonucleases *xrn2 xrn3* that are thought to be downstream effectors of this pathway (Estavillo et al., 2011). In fact, the expression changes triggered in the *xrn2 xrn3* mutants showed the highest similarity to antimycin A treatment, *atphb3* and *letm1(*−/−*)* *Letm2(*+/−*)* mutation, providing evidence for a role of PAP signaling in mitochondrial retrograde regulation. Msh1 was recently shown to be dual-targeted to mitochondria and chloroplasts, but based on the expression pattern the effects appear to be more similar to mitochondrial perturbations. This may be because RecA3 is thought to be specifically mitochondrial (Xu et al., 2012). Finally, Group III contained the mutations or treatments that affect chloroplast function, such as inhibitors norflurazon and lincomycin which resulted in similar responses, as well as PSII inhibitor PNO8 and the *flu* mutant. The chloroplast mutants in S-sulfocysteine synthase *cs26*, ferredoxin NADH-oxidoreductase *fnr1* and triose-phosphate translocator *tpt-1* also clustered in Group III. Group III also contained the mitochondrial inhibitors oligomycin (ATP synthase) and rotenone (Complex I), suggesting that these inhibitors also inhibit chloroplast ATP synthase and a multi-subunit NADH dehydrogenase complex that has been characterized on the thylakoid membrane (Ifuku et al., 2011).

### Identification of marker genes for energy organellar dysfunction

Next, genes that consistently respond to defects in mitochondrial and/or chloroplast functions were identified. Of 22810 ATH1 probe sets, 136 were significantly changed [twofold change with PPDE (<*p*) > 0.95] in at least six mitochondrial perturbations, while 60 probe sets were significantly changed in at least six chloroplast perturbations (Table S1 in Supplementary Material). Subsequently, 14 probe sets were identified that are changed in more than six mitochondrial and chloroplast perturbations and can thus be considered as general markers for mitochondrial and chloroplast dysfunction (Table 4). Conversely, a comparison was made to identify genes that strictly respond to either mitochondrial or chloroplast perturbation (significantly changed in seven or more mitochondrial and six or more chloroplast perturbations) but not the other (significantly changed in 0 or 1 perturbation). Twelve probe sets were identified that respond to mitochondrial but not chloroplast perturbation (Table 4), including *At5g09570* encoding a putative mitochondrial protein of unknown function that was significantly changed in 10 mitochondrial perturbations but in 0 chloroplast perturbations. Thus, *At5g09570* is an excellent marker gene to predict mitochondrial dysfunction. In contrast, 14 probe sets responded to chloroplast perturbations but not to mitochondrial stresses (Table 4), including a DNAJ heat shock protein that responded to seven chloroplast perturbations and only one mitochondrial perturbation (oligomycin), and a probe detecting Toll/Interleukin type receptors *At1g72910* and *At1g72930* responding to seven chloroplast but 0 mitochondrial perturbations.

**Table 4:** **Marker genes for mitochondrial and/or chloroplast perturbations**.

Probe ID	Mitoch. perturb.	Chloropl. perturb.	AGI Code	Description	Loc.
260522_x_at	11	7	AT2G41730	Unknown protein	c
264042_at	10	7	AT2G03760	AtST1 sulfosteroid transferase	c
263402_at	10	6	AT2G04050	MATE efflux family protein	pm
263515_at	10	6	AT2G21640	UPOX	m
253046_at	9	6	AT4G37370	CYP81D8 cytochrome P450	er
247655_at	7	6	AT5G59820	ZAT12 transcription factor	n
263231_at	7	6	AT1G05680	UGT74E2 IBA UDP-glucosyl transferase	c
245038_at	6	7	AT2G26560	Phospholipase 2A PLP2	c
263403_at	6	7	AT2G04040	ATDTX1 MATE transporter	pm
246099_at	6	6	AT5G20230	AtBCB Blue-Copper binding protein	pm
247949_at	6	6	AT5G57220	CYP81F2 cytochrome P450	er
252131_at	6	6	AT3G50930	BCS1 AAA-type ATPase family protein	m
256376_s_at	6	6	AT1G66690;AT1G66700	SAM:carboxyl methyltransferase	c
259479_at	6	6	AT1G19020	Unknown protein	c
250515_at	10	0	AT5G09570	Unknown protein AtMSM1	p, m
253322_at	8	1	AT4G33980	Unknown protein	n
267036_at	8	1	AT2G38465	Unknown protein	pm
267364_at	8	0	AT2G40080	ELF4 (EARLY FLOWERING 4)	n
248709_at	7	0	AT5G48470	Unknown protein	n
252563_at	7	1	AT3G45970	Expansin ATEXLA1	ec
254208_at	7	0	AT4G24175	Unknown protein	p
256060_at	7	1	AT1G07050	CONSTANS-like protein	n
256252_at	7	0	AT3G11340	UGT76B1 glycosyltransferase	pm
263483_at	7	0	AT2G04030	Heat shock protein 90.5 kDa	p
267237_s_at	7	0	AT2G44040; AT3G59890	Dihydrodipicolinate reductase family	p, m
267474_at	7	0	AT2G02740	ATWHY3 transcription factor	p, m
256999_at	1	7	AT3G14200	DNAJ heat shock protein	n
262374_s_at	0	7	AT1G72910; AT1G72930	Disease resistance protein TIR-NBS	c
245876_at	1	6	AT1G26230	Chaperonin TCP-1	p
246313_at	1	6	AT1G31920	Pentatricopeptide (PPR) repeat- protein	c
246792_at	1	6	AT5G27290	Unknown protein	p
250054_at	1	6	AT5G17860	CAX7 calcium exchanger	pm
251218_at	1	6	AT3G62410	CP12-2	p
253104_at	1	6	AT4G36010	Pathogenesis-related thaumatin family	ec
253971_at	1	6	AT4G26530	Fructose-bisphosphate aldolase	p
256015_at	1	6	AT1G19150	LHCA6 PSI light-harvesting complex	p
260266_at	1	6	AT1G68520	Zinc finger (B-box type) family protein	n
261118_at	1	6	AT1G75460	ATP-dependent LON protease	p
264774_at	1	6	AT1G22890	Unknown protein	ec
266279_at	1	6	AT2G29290	Tropinone reductase	c

*Top: list of probe sets that respond to at least six experiments affecting mitochondrial and chloroplast perturbations. Middle: list of probe sets that respond in at least six experiments affecting mitochondrial function but not chloroplast function. Bottom: list of probe sets that respond in at least six experiments that affect chloroplast function but not mitochondrial function. Abbreviations: Loc, subcellular location; p, plastid; m, mitochondrial; pm, plasma membrane; ec, extracellular; er, endoplasmic reticulum; n, nucleus; c, cytosol. Underlined locations indicated experimental evidence*.

**Figure 3:**
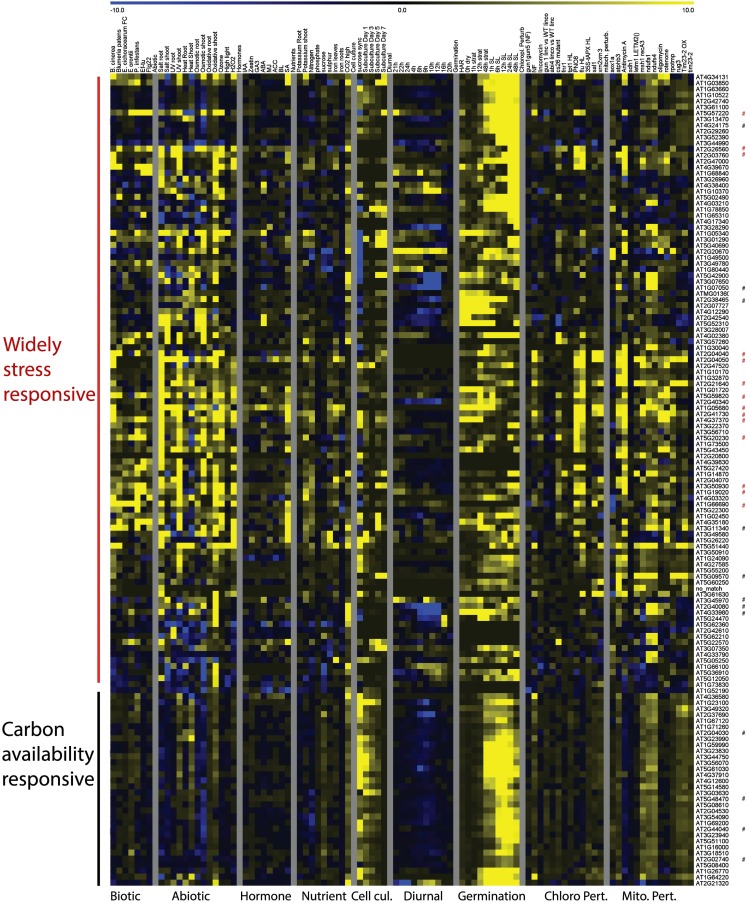
**Heat Map representing expression values of genes commonly responding to mitochondrial perturbations**. Normalized expression values for genes responding to at least 6 of 13 experiments relating to mitochondrial perturbations in a variety of stress and development related microarray experiments. Red asterisks indicate probe sets that were also commonly altered by chloroplast perturbations, black asterisks indicate probe sets that were specifically responding to mitochondrial perturbations. Abbreviations: Cell cul, cell culture; Developm, development; Germin, germination; Chloro Pert, chloroplast perturbation; Mito pert, mitochondrial perturbation.

The expression patterns of the genes most widely responsive to mitochondrial or chloroplast perturbations were also analyzed during a variety of environmental perturbations, including biotic and abiotic stresses, hormone treatments and nutrient deprivations, diurnal cycle, germination, cell cycle progression and plant developmental stages and tissue-types. Normalized expression values were hierarchically clustered to investigate expression patterns. Expression analysis of the genes responding to six or more mitochondrial perturbations revealed they were clearly divided into two main groups, with the largest group being induced not only by mitochondrial perturbations, but also by biotic and abiotic stresses, nutrient deficiencies, and in terms of hormone treatments mostly by salicylic acid (Figure 3).

This indicates that these genes are likely to be part of the general cellular stress signaling pathways. The 14 probe sets that respond to both mitochondrial and chloroplast perturbations are all part of the general stress-responsive cluster (Figure 3).

**Figure A1:**
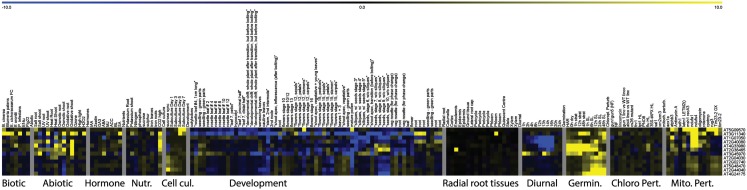
**Heat Map representing expression values of genes specifically responding to mitochondrial perturbations**. Normalized expression values for genes responding to at least seven of 13 experiments relating to mitochondrial perturbations and 0 or 1 chloroplast perturbations in a variety of stress and development related microarray experiments.

A second group of genes was far less responsive to external stress conditions and appeared to be most strongly altered by mitochondrial perturbations. Moreover, these genes showed a clear peak in expression shortly after the addition of sucrose or fresh nutrient media to stagnating suspension cells and under high CO_2_ concentrations, linking these genes more directly with carbon availability and the basic energy-supply function of mitochondria. The genes in this second group showed clear induction in the later time points of stratification in the seed germination experiment and furthermore showed a dip in expression shortly after the start of daylight during the diurnal cycle. The expression patterns of the 12 genes that were responding to mitochondrial but not chloroplast stresses were varied and were spread over the generally stress-responsive and carbon availability responsive groups (Figure 3; Figure A1 in Appendix). For instance the *At5g09570* gene showed very high foldchanges (e.g., 67 fold in the *atphb3* mutant) in response to mitochondrial perturbations, but was relatively stably expressed in all of the other tissues, developmental stages and showed only mild stress-inducible expression (sixfold during salt stress). Four of the other genes were strongly downregulated during the diurnal cycle with a low-point around the onset of day including circadian clock related *COL9* Constans-like protein and *Early Flowering 4*. A gene *At3g45970* encoding an expansin-like protein peaked at both day-night transition points, whereas the expression of five genes appeared to be linked with green tissues and are induced once light signals are received during germination (Figure A1 in Appendix). In conclusion, it appears that the major trends for response to mitochondrial perturbation are induction of a general cell-wide stress response, and the induction of multiple genes that are induced by high carbon and carbohydrate supply.

**Figure 4:**
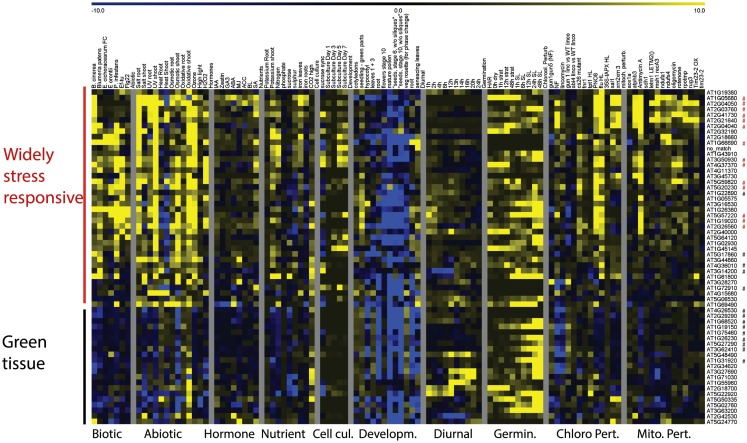
**Heat Map representing expression values of genes commonly responding to chloroplast perturbations**. Normalized expression values for genes responding to at least 6 of 13 experiments relating to chloroplast perturbations in a variety of stress and development related microarray experiments. Red asterisks indicate probe sets that were also commonly altered by mitochondrial perturbations, black asterisks indicate probe sets that were specifically responding to chloroplast perturbations.

Clustering of expression patterns for the genes responding to chloroplast perturbations revealed a similar division into two clusters (Figure 4). The largest cluster contained genes that were broadly induced by chloroplast perturbations, general stresses and nutrient deficiencies, and contain the genes that are in common with mitochondrial perturbations. Conversely, the second group consist of genes of which the majority is downregulated by lincomycin and norflurazon, linking them with the classical GUN-mediated chloroplast retrograde pathways of chlorophyll synthesis inhibition (Koussevitzky et al., 2007). Interestingly, these genes tend to be downregulated by environmental stresses as well, as opposed to the first, stress-induced, cluster. When looking at their expression patterns during development and in different tissue-types, the genes of the second cluster are consistently expressed most highly in green tissues and less in roots, pollen, late flowers and seeds, and their expression usually increases during germination as soon as light signals are received. In contrast, the stress-induced cluster shows more random expression across tissues and development. Most of the genes that respond to chloroplast but not mitochondrial dysfunction belong to the second cluster containing genes that are downregulated during chlorophyll biosynthesis inhibition, general stresses and in non-green tissues. Therefore it seems that at least two types of chloroplast retrograde pathways are at play, one directly linked with the functioning of chloroplasts in photosynthetic conditions, and a second pathway which triggers a more general cellular stress response that is at least partly in common with mitochondrial perturbation signaling.

To obtain insight into the regulatory context of the identified marker genes, a promoter analysis was performed to identify the presence of known transcription factor binding motifs using the Athena promoter analysis tool (Table 5). Several statistically overrepresented motifs were found in the different marker categories. For marker genes responding to both mitochondrial and chloroplast perturbations overrepresentation was found for W-boxes (WRKY-binding site, *p*-value 0.0054; Yu et al., 2001), T-boxes (*p*-value 0.0137; Chan et al., 2001), and the CACGTG motif (G-box/ABA response element ABRE) which is known to bind both light responsive and ABA responsive transcription factors (*p*-value 0.0307; Guiltinan et al., 1990; Menkens and Cashmore, 1994). In the promoters of marker genes for mitochondrial perturbations putative MYB transcription factor binding sites (*p*-value 0.02530) and Evening Elements involved in circadian regulation (*p*-value 0.0357; Harmer et al., 2000) were overrepresented. Interestingly the three genes that contain the Evening Elements are strongly reduced in expression early in the day (Figure 3; Figure A1 in Appendix). Thirdly, in the promoters of marker genes for chloroplast perturbations the I-box element involved in light signaling (*p*-value 0.0152; Giuliano et al., 1988), a GCCGCC motif responding to ethylene (*p*-value 0.0345; Solano et al., 1998) and also ABA response elements (*p*-value 0.0365) were overrepresented. Overall, it appears that WRKY transcription factors are involved in coordinating the common aspects of transcriptional responses to mitochondrial and chloroplast perturbations indicated by the highly significant overrepresentation and wide occurrence of W-boxes. Also the overrepresentation and wide occurrence of the I-box involved in light signaling is likely to be important in the regulation of many of the chloroplast proteins.

**Table 5:** **Occurrence of known transcription factor binding sites in marker genes**.

**MITOCHONDRIAL AND CHLOROPLAST PERTURBATIONS**				
AGI Code	Description	W-box	T-box	CACGTG
AT2G41730	Unknown protein	3	1	
AT2G03760	AtST1 sulfosteroid transferase	2	1	
AT2G04050	MATE efflux family protein	1		1
AT2G21640	UPOX	2		1
AT4G37370	CYP81D8 cytochrome P450	3	2	1
AT5G59820	ZAT12 transcription factor	1	1	
AT1G05680	UGT74E2 IBA UDP-glucosyl transferase	1	1	1
AT2G26560	Phospholipase 2A PLP2	3	2	
AT2G04040	ATDTX1 MATE transporter	3	1	
AT5G20230	AtBCB Blue-Copper binding protein	1	1	
AT5G57220	CYP81F2 cytochrome P450	2	4	
AT3G50930	BCS1 AAA-type ATPase family protein	2	1	
AT1G66690	SAM:carboxylmethyl transferase		1	1
AT1G19020	Unknown protein	3		
**MITOCHONDRIAL PERTURBATIONS**				
AGI Code	Description	Evening Element	MYB	
AT5G09570	Unknown protein AtMSM1			
AT4G33980	Unknown protein	1		
AT2G38465	Unknown protein			
AT2G40080	ELF4 (EARLY FLOWERING 4)	1		
AT5G48470	Unknown protein			
AT3G45970	Expansin ATEXLA1		1	
AT4G24175	Unknown protein			
AT1G07050	CONSTANS-like protein	1	1	
AT3G11340	UGT76B1 glycosyltransferase			
AT2G04030	Heat shock protein 90.5 kDa		1	
AT2G44040	Dihydrodipicolinate reductase family		1	
AT2G02740	ATWHY3 transcription factor			
**CHLOROPLAST PERTURBATIONS**				
AGI Code	Description	I-box	GCCGCC	ABRE
AT3G14200	DNAJ heat shock protein		1	1
AT1G72910	Disease resistance protein TIR-NBS	1	1	
AT1G26230	Chaperonin TCP-1			
AT1G31920	Pentatricopeptide (PPR) repeat- protein	1		
AT5G27290	Unknown protein	1		
AT5G17860	CAX7 calcium exchanger			
AT3G62410	CP12-2	1		
AT4G36010	Pathogenesis-related thaumatin family		1	
AT4G26530	Fructose-bisphosphate aldolase	1		
AT1G19150	LHCA6 PSI light-harvesting complex			
AT1G68520	Zinc finger (B-box type) family protein	3		
AT1G75460	ATP-dependent LON protease	1		1
AT1G22890	Unknown protein	1		
AT2G29290	Tropinone reductase	1		

*The 1 kb upstream promoter region of the marker genes for mitochondrial and/or chloroplast perturbations were searched for known transcription factor binding motifs. Statistically overrepresented motifs are shown for the different marker genes. The number of occurrences of each element per promoter are shown*.

**Figure 5:**
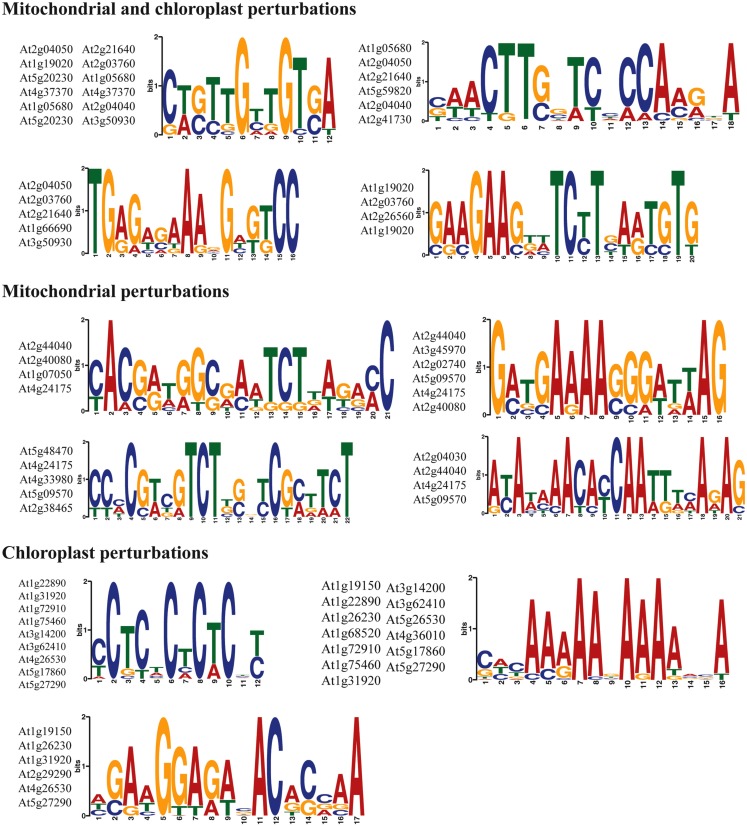
**Discovery of sequence motifs in the promoters of marker genes for retrograde regulation**. The 1 kb upstream promoter regions were searched for common motifs using the MEME suite. Sequence consensus logos and the genes in which the promoter motifs occurred are shown.

Finally, the 1 kb upstream promoter regions of the top marker genes (Table 4) were analyzed for common sequence motifs using the MEME discovery suite (Bailey et al., 2009). A number of putative sequence motifs for each of the promoter sets were discovered and these are represented in Figure 5. Additional work will be required to validate their function in the regulation of retrograde responses, for instance using promoter reporter studies and identification of binding factors by yeast one-hybrid screens.

## Discussion

In this study we aimed to compare genome-wide transcriptional responses to perturbations of mitochondrial or chloroplast function. Retrograde signaling for both chloroplasts and mitochondria has classically been studied using chemical inhibitors such as norflurazone, lincomycin, rotenone, and antimycin A. To get a broader picture several mutants in specific mitochondrial or chloroplast proteins were also included in this study. One of the interesting outcomes of this study is the surprisingly high degree of similarity between mitochondrial and chloroplast retrograde responses to functional perturbations. In fact, over a quarter of the genes that regularly respond to chloroplast perturbations can also respond to mitochondrial perturbations. When looking at the functional categories that are common between the two, prominent categories are stress response, redox balance, transport, cell wall composition, starch metabolism but also auxin, brassinosteroid, and salicylic acid metabolism. In addition WRKY transcription factors appear to be on the interface between chloroplast and mitochondrial perturbations, making them interesting candidates for further characterization. In agreement, an overrepresentation of putative TTGAC WRKY-binding motifs was noted in the promoters of several highly stress-responsive genes encoding mitochondrial proteins (Van Aken et al., 2009). Differences between the two responses are related to the two basic functions of each organelle and their maintenance, namely photosynthesis and respiration. This is also reflected in the specific overrepresentation of genes encoding proteins that are targeted to the organelle that is primarily affected (Table 2), but not the other two energy organelles. In other words this analysis statistically demonstrates that organellar retrograde regulation ‘works’ in the sense that it is able to discriminate the source of the signal despite vastly overlapping signals.

The observation that the top overlapping genes are highly responsive to general abiotic and often biotic stresses raises the question whether this subset of stress-responsive genes is actually responding to organellar dysfunction. At least two scenarios can be envisaged, one in which general stresses and organellar dysfunction trigger the same signaling pathway resulting in the altered expression of these genes. In another scenario, the genes are directly responding to organelle function, and general stresses lead to impairment of these organelles, which thus results in changes in their expression. The fact that a set of 30 genes, almost all part of the top common responsive genes between chloroplast and mitochondrial perturbations, were previously reported to be uniquely altered in expression in an overexpression line of the mitochondrial protein AtPHB4, supports the second scenario (Van Aken et al., 2007, 2010). The observation that this gene set can be completely uncoupled from the general stress response suggests that a part of the response to mitochondrial, but also chloroplast dysfunction and general stress response is routed through the mitochondria. The exact signaling intermediates and transcription factors involved are thus far elusive. Another interesting finding from this study is that gene expression changes triggered by the recently uncovered chloroplast retrograde pathway involving PAP as a putative signaling molecule, PAP phosphatase SAL1 and the XRN-type exoribonucleases that are inhibited by PAP (Estavillo et al., 2011), shows highest similarity to mitochondrial perturbations. Similarity appears to be largest with the dataset for antimycin A and a number of mitochondrial mutants such as *atphb3*, *msh1 recA3*, and *letm1 LETM2(*+/−*)*. This leads to the hypothesis that the stress signal that is routed through the mitochondria might in fact be PAP, further corroborated by the fact that SAL1 is dual-targeted to chloroplasts and mitochondria.

Several marker genes have been identified in this study that may be useful for screening for organellar dysfunction (Table 4). In the marker genes that respond commonly to mitochondrial and chloroplast perturbations a number have been previously characterized to be involved in hormone metabolism: *UGT74E2* encodes an oxidative stress-responsive indole-butyric acid (IBA) glycosyltransferase involved in shoot branching and osmotic stress tolerance (Tognetti et al., 2010), and brassinosteroid sulfotransferase *AtST1* (Marsolais et al., 2007). Other genes also appear to be involved in enzymatic conversion or transport of chemicals: two cytochrome P450 oxygenating enzymes CYP81F2 and CYP81D8 belonging to the same family, methyltransferase PXMT1 that methylates 1,7-paraxanthine (or its homolog At1g66690) and 2 MATE-type efflux carrier proteins. Several proteins have also been reported to be involved in oxidative stress tolerance and cell death such as blue-copper binding protein AtBCB (Ezaki et al., 2005), UGT74E2 (Tognetti et al., 2010), and phospholipase PLP2 (La Camera et al., 2009). Also, the zinc finger transcription factor ZAT12 has been shown to be an important player in regulating the transcriptome in response to abiotic (e.g., cold), oxidative stresses (Rizhsky et al., 2004; Davletova et al., 2005; Vogel et al., 2005), and high-light (Iida et al., 2000), thus it will be of interest to investigate if ZAT12 is also involved in executing retrograde responses. Finally, a number of genes of unknown function that have previously been studied in other stress-related studies were retained including *UPOX*, *BCS1*, *At2g41730* (Gadjev et al., 2006; Ho et al., 2008; Van Aken et al., 2009), and *At1g19020*.

Looking at the 12 potential marker genes that respond uniquely to mitochondrial perturbations there is a striking number of genes encoding proteins of unknown function. The most interesting marker gene *At5g09570* responds to 10 of 14 mitochondrial experiments and no chloroplast experiments. It contains a CHCH (coiled coil – helix – coiled coil – helix) domain and shows homology with the COX19 protein from yeast that is important for post-translational processing of mitochondrial cytochrome c oxidase (Nobrega et al., 2002). *At5g09570* is also predicted to be mitochondrially targeted, so therefore we propose the name *AtMSM1* for *A. thaliana Mitochondrial Stress Marker 1*. *At4g24175* contains a domain found in bacterial protein of unknown function YjgA that co-migrates with the 50S ribosomal particle. Genes that have been characterized include *AtWHY3* encoding a DNA-binding protein that was shown to be involved in organellar genome stability (Marechal et al., 2009). Interestingly, AtWHY3 was shown to be located to plastids. AtWHY2 is active in the mitochondria and its transcript *At1g21760* also responds in six mitochondrial but none of the chloroplast perturbations, making it a good marker gene as well (Figure 3). The Whirly proteins bind ssDNA to promote accurate repair of DNA double-strand breaks (Cappadocia et al., 2010). The isoleucine glycosyltransferase*UGT76B1* functions on the interface between SA and JA-mediated pathogen resistance (Von Saint Paul et al., 2011). Surprisingly, three genes encoding proteins closely related to circadian rhythm were responsive to mitochondrial perturbations, *Early Flowering 4* being a phytochrome-regulated negative regulator of the central clock oscillator (Kikis et al., 2005), a Constans-like gene *At1g07050* and *Expansin ATEXLA1*. It has previously been recognized that leaf expansion is affected by day and night transitions, possibly in relation to starch content. This may point toward a role of mitochondria and energy status in influencing the diurnal cycle (Pantin et al., 2011). Furthermore, a 90.5 kDa heat shock protein was identified that is targeted to plastids and has also been identified in mitochondria (Cao et al., 2003; Ito et al., 2006). In another study, TCP transcription factors were shown to link expression of genes encoding mitochondrial proteins with the diurnal cycle through the presence of site II promoter elements (Giraud et al., 2010). Together, these results suggest that mitochondrial function is regulated by the diurnal cycle and that in turn mitochondria may provide feedback, likely about energy status, to the central clock regulators.

Finally, a number of genes were identified that specifically respond to chloroplast perturbations but not mitochondrial perturbations (Table 4; Figure 4). The list contains several genes encoding plastid proteins: light-harvesting complex subunit *LHCA6*, chaperonin *TCP-1*, a LON protease, the CP12-2 stromal peptide found in protein complex with glyceraldehyde-3-phosphate dehydrogenase (GAPDH) and phosphoribulokinase (PRK) embedded in the Calvin cycle, an unknown protein *At5g27290* and fructose-bisphosphate aldolase *ATFBA5*. Hence, these genes might be useful markers for future chloroplast retrograde signaling studies. Several other genes encoding proteins with a variety of functions are present including a DNAJ chaperonin, a PPR protein *At1g31920*, calcium exchanger 7, NAD(P) tropinone oxidoreductase *At2g29290*, a pathogenesis-related thaumatin and a Toll/Interleukin-like receptor. Interestingly, an uncharacterized B-box Zinc Finger transcription factor is also specifically induced by chloroplast perturbations.

In conclusion, this meta-analysis of mitochondrial and chloroplast retrograde signaling has revealed novel insights into the strong similarities between the two pathways. To a large extent these overlapping signals are in common with more general abiotic and biotic stress-responses. Our results suggest that in the case of general stresses a subset of genes may indeed be responsive to functional perturbation of the energy organelles that are caused by the stress conditions. Nevertheless, specific responses can be found for each organelle and a number of novel marker genes have been identified for further functional characterization and use in screening methods. Furthermore, the results suggest that the recently identified chloroplast retrograde pathway involving PAP is likely to be closely linked to mitochondrial function as well and is a potential candidate for triggering the organelle-induced stress-responses in general. Future research will be required to fully understand the role of PAP and its associated proteins in retrograde signaling.

## Conflict of Interest Statement

The authors declare that the research was conducted in the absence of any commercial or financial relationships that could be construed as a potential conflict of interest.

## Supplementary Material

The Supplementary Material for this article can be found online at http://www.frontiersin.org/Plant_Physiology/10.3389/fpls.2012.00281/abstract

## Footnotes

^1^http://www.peroxisome.msu.edu

^2^www.tm4.org/mev

^3^www.arabidopsis.org
